# NutriChem: a systems chemical biology resource to explore the medicinal value of plant-based foods

**DOI:** 10.1093/nar/gku724

**Published:** 2014-08-08

**Authors:** Kasper Jensen, Gianni Panagiotou, Irene Kouskoumvekaki

**Affiliations:** 1 Center for Biological Sequence Analysis, Department of Systems Biology, Technical University of Denmark, Kemitorvet, Building 208, DK-2800 Lyngby, Denmark; 2 School of Biological Sciences, The University of Hong Kong, Pokfulam Road, Hong Kong

## Abstract

There is rising evidence of an inverse association between chronic diseases and diets characterized by rich fruit and vegetable consumption. Dietary components may act directly or indirectly on the human genome and modulate multiple processes involved in disease risk and disease progression. However, there is currently no exhaustive resource on the health benefits associated to specific dietary interventions, or a resource covering the broad molecular content of food. Here we present the first release of NutriChem, available at http://cbs.dtu.dk/services/NutriChem-1.0, a database generated by text mining of 21 million MEDLINE abstracts for information that links plant-based foods with their small molecule components and human disease phenotypes. NutriChem contains text-mined data for 18478 pairs of 1772 plant-based foods and 7898 phytochemicals, and 6242 pairs of 1066 plant-based foods and 751 diseases. In addition, it includes predicted associations for 548 phytochemicals and 252 diseases. To the best of our knowledge this database is the only resource linking the chemical space of plant-based foods with human disease phenotypes and provides a foundation for understanding mechanistically the consequences of eating behaviors on health.

## INTRODUCTION

The term ‘exposome’, used to describe the totality of all environmental exposures (e.g. diet, air pollutants, lifestyle factors) over the life course of an individual, has been proposed as a critical entity for disease etiology, complementary to genetic predisposition (1–3). In order to avoid biased inferences regarding gene-environment interactions and to discover the major causes of chronic diseases, a more comprehensive and quantitative view of the exposome is required (4,5). Since a full characterization of the human exposome is a daunting task, cutting the pie to smaller pieces could offer critical portions of disease associations to certain exposures.

**Figure 1.**
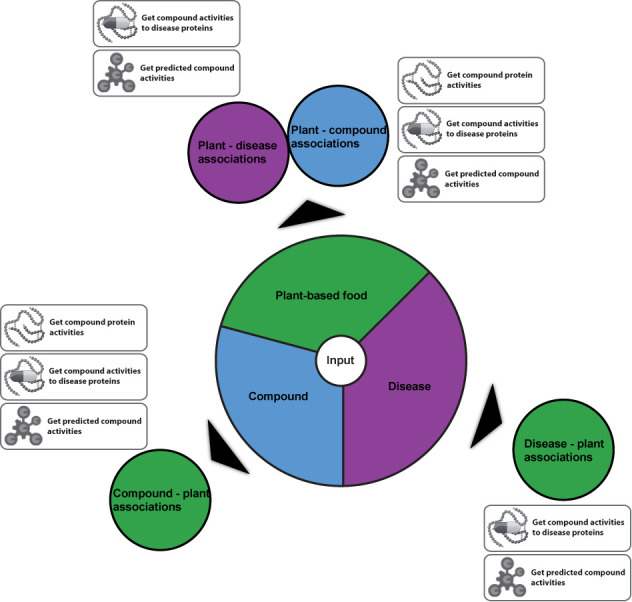
The different functionalities of NutriChem. The user can query NutriChem by food, disease or compound name/ID. Outcomes from each query type are in circles indicated by arrows. The available actions that can be subsequently performed are depicted next to each circle.

Diet is certainly one of the most dynamic expressions of the exposome and one of the most challenging to assess its effects in health homeostasis and disease development, considering its myriad components and their temporal variation. It is estimated that almost 80% of chronic diseases could be avoided by consumption of healthier food, whereas meta-analysis of observational studies has shown a dose-response effect of fruits and vegetables on cardiovascular diseases and stroke risk (6–12). Recognizing, understanding and interpreting the interplay between diet and biological responses may contribute to the weight of evidence in assigning causality to a diet-disease association. Therefore, in order to open up new avenues to disease prevention through diet interventions, it is crucial to provide insights into the mechanisms by which the chemical space of food constituents might be exerting its effects. Toward this direction we have developed a state-of-the art database with information on plant-based food (referred to simply as ‘food’ throughout the article), its small compound constituents (also known as phytochemicals) and human disease phenotypes associated with it (Figure 1). This database offers a unique platform for exploring the medicinal value of diet and elucidating the synergistic effects of natural bioactive compounds on disease phenotypes.

There are several ongoing efforts, though rather limited in focus and size, which aim to collect information in a single resource regarding the molecular composition of food, i.e. the Danish Food Composition Database (http://www.foodcomp.dk) centered on well-known organic nutrients, such as vitamins, amino acids, carbohydrates and fatty acids; the Phenol-Explorer (13) with information in text format for 500 polyphenols in over 400 foods and the KNApSAcK Family Database (14). For a molecular systems chemical biology approach of diet, the lack of chemical structures and high-throughput retrieval of data in the above databases are significant bottlenecks for linking food, its phytochemical content and related biological activities in a straightforward process. Likewise, the MAPS database (http://www.mapsdatabase.com/) that has a scope very similar to NutriChem, does not use detailed disease ontology and includes information from only 300 papers extracted manually from PubMed.

## IMPLEMENTATION

### Data ontologies

The taxonomy of the plant species was retrieved from NCBI taxonomy (http://www.ncbi.nlm.nih.gov/taxonomy). Food names were retrieved from the Plant For A Future (PFAF) (http://www.pfaf.org) and the Danish Food Composition Databank (http://www.foodcomp.dk) and were mapped to NCBI IDs (TAXIDs). Human proteins were associated to diseases through the Therapeutic Targets Database (15) (TTD Version 4.3.02). Disease names were mapped to the OBO Foundry Human Disease Ontology (DOID) (16) and ordered in disease categories. Chemical structures and corresponding IDs of small compounds were retrieved from PubChem (17) ChEBI (18), CHEMLIST (19), the Chinese Natural Product Database (20) (CNPD) and Ayurveda (21) resources. Marvin 6.1.0 (http://www.chemaxon.com) was used for encoding the chemical structures in unique SMILES.

### Food-compound and food-disease associations

We extracted by text-mining plant—phytochemical and plant-disease associations from 21 million abstracts in PubMed/MEDLINE, covering the period 1908–2012, as described previously (22). A Naive Bayes Classifier (https://pypi.python.org/pypi/NaiveBayes) was trained to recognize pairs of plants and phytochemicals, in the one case, and pairs of plants and diseases in the other. The performance of the classifier was evaluated in each case on an external, balanced test set of 250 positive and negative abstracts, and resulted to 88.4% accuracy and 87.5% F1-measure, and 84.5% accuracy and 84.4% F1-measure, respectively. We subsequently filtered for pairs involving plant-based foods using PFAF (http://www.pfaf.org) and the Danish Food Composition Databank (http://www.foodcomp.dk). In total, NutriChem contains 18478 pairs of 1772 plant-based foods and 7828 phytochemicals, and 6242 pairs of 1066 plant-based foods and 751 diseases.

### Association of diet to health benefit at molecular level

Fisher's exact test was used to systematically associate frequently occurring phytochemical-disease pairs through the phytochemical-food and food-disease relations extracted by text mining, with the Benjamini–Hochberg procedure and a 5% false discovery rate (the method has been described in detail previously (22)). Chemical–protein interactions data were gathered in September 2013 from the open-source database ChEMBL (version 16). We associated phytochemicals that have no direct experimental bioactivity data with structurally similar compounds from ChEMBL (Tanimoto coefficient > 0.85) and their protein targets, when such data were available. For the calculation of the Tanimoto coefficient, chemical structures were encoded in 166 MACCS keys (23) using OpenBabel (24). In total, NutriChem contains 1549 predicted associations between 548 phytochemicals and 252 diseases.

### Visual interface

We implemented a visual interface in NutriChem to facilitate the visualization of the results using CytoscapeWeb (http://cytoscapeweb.cytoscape.org). At the left part of the screen a network is depicted, with the query input as the central node and the retrieved results connected to it through edges. The thickness of an edge indicates the number of references in support of an association. By clicking on the icon ‘Apply layout’ the user can apply the force-directed layout on the network. At the right-hand side the results are shown as a list. The list items are expandable upon click and detailed information about the association is shown. The list items are also expandable by clicking on the edges of the network at the left-hand side. By clicking on the icon ‘Export network’ the user can download the network in Cytoscape/xml format and continue working offline in Cytoscape or download the list results as a ‘tab-separated file’ by clicking on the ‘Export table’ icon. The user can search by: (a) food name or TAXID, (b) human disease name or DOID and (c) compound (i.e. phytochemical) name, ID (ChEMBL ID, CID, etc.) or SMILES string.

## APPLICATIONS

### Food as query

**Figure 2.**
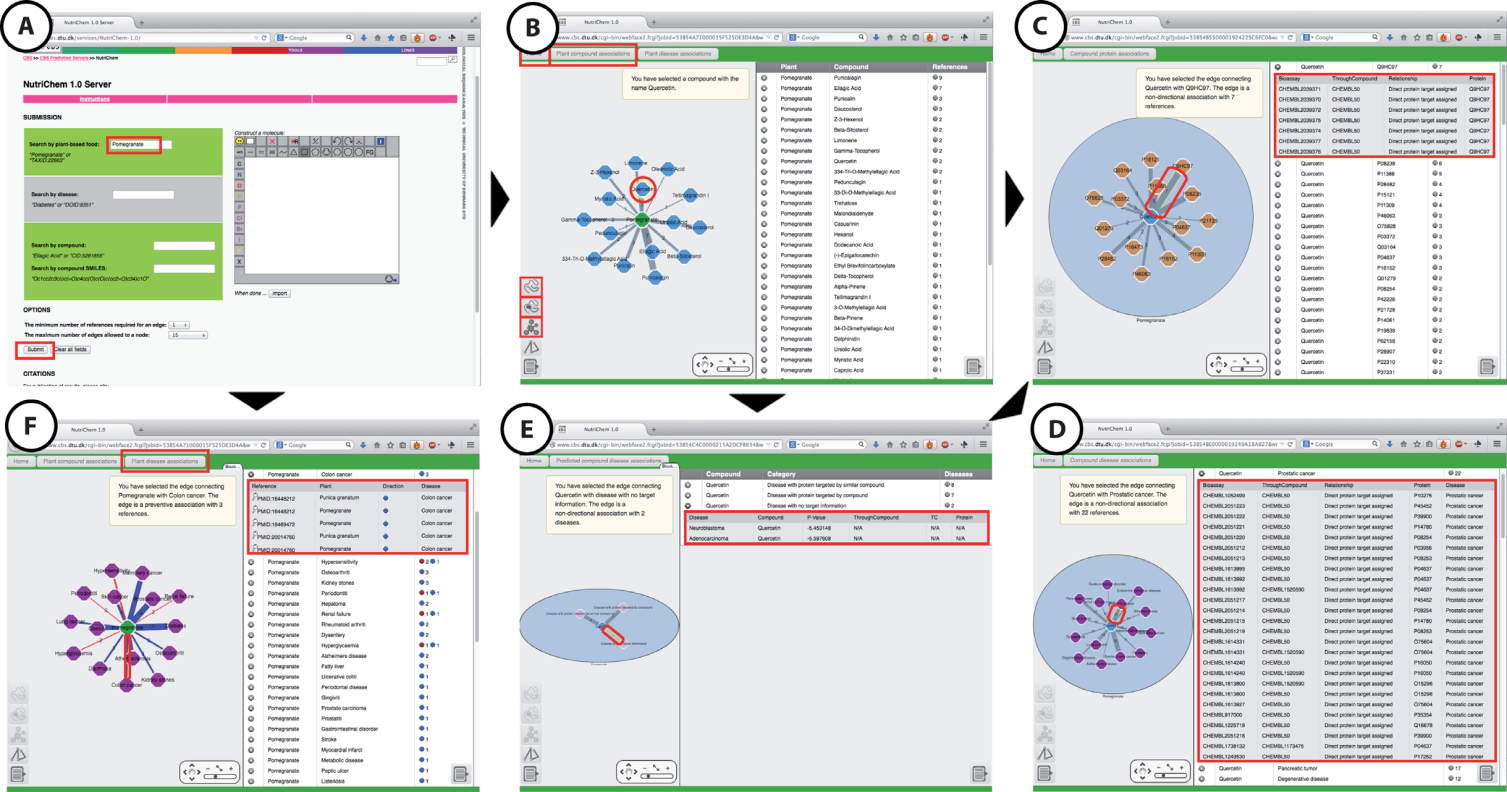
(**A**) A search with ‘pomegranate’ as the query, (**B**) The plant-compound network is shown by default and by using the top buttons the user can switch to the plant-disease network or return to Home for submitting a new query. On the right-hand side the user can directly access all relevant references in PubMed in support of the pomegranate-compound associations, by clicking on the respective PMID. (**C**) If we select the compound ‘quercetin’, ‘Get compound protein activities’ creates a network of 15 proteins, by default, with measured experimental activity. On the complete result list on the right side of the panel, the user has the option to click either on the bioassay, the compound, or the protein IDs, which will open in a new tab the respective ChEMBL and UniProt pages. (**D**) ‘Get compound activities to disease proteins’ filters the above results for therapeutic proteins only (proteins annotated to a disease in TTD) and displays the disease-associated network for quercetin. (**E**) ‘Get predicted compound activities’ shows predicted disease associations for quercetin as derived from Fisher's test. The results are grouped in three categories, depending on the experimental evidence that supports each prediction. (**F**) The disease network of the query ‘pomegranate’ returns by default 15 diseases in which pomegranate is known to have an effect. Blue edge: preventive association, red edge: promoting association. On the right-hand side the user can directly access all relevant references in PubMed in support of the pomegranate-disease associations, by clicking on the respective PMID.

When a food query is submitted to the server, the user can specify the minimum number of references required for an edge and the maximum number of edges allowed to a query node. By default we use a limit of minimum one reference for an edge (can range from 1 to 10) and maximum 15 edges for a central node (can range from 1 to 20). However, regardless of the settings, all results are listed at the right-hand side. Figure 2 illustrates a search with ‘pomegranate’ as the query. In the top of the interface three buttons are shown. The buttons allow the user to switch between the ‘Home’ page and the two different result sections. The plant-compound associations network is shown by default and by using the top buttons the user can switch to the plant-disease network or return to Home for submitting a new query. The network at the left hand-side shows, by default, 15 compound associations. On the right-hand side the user can directly access all relevant references in PubMed in support of the pomegranate-compound associations, by clicking on the respective PubMed Identifier (PMID).

When a compound node is selected on the food-compound network, the user has three special action buttons: ‘Get compound protein activities’, ‘Get compound activities to disease proteins’ and ‘Get predicted compound activities’. For example, if we select the compound ‘quercetin’, ‘Get compound protein activities’ lists proteins in UniProtID (http://www.uniprot.org) with measured experimental activity (data from ChEMBL). The user has the option to click either on the bioassay, the compound, or the protein IDs on the right side of the panel, which will open in a new tab the respective ChEMBL and UniProt pages. ‘Get compound activities to disease proteins’ filters the above results for therapeutic proteins only (proteins annotated to a disease in TTD) and displays the disease-associated network for quercetin. ‘Get predicted compound activities’ shows predicted compound-disease associations as derived from Fisher's test. The results are grouped in three categories, depending on the experimental evidence that supports each prediction. The first category ‘Disease with protein targeted by compound’ includes predicted disease associations, where there exist experimental activity data for quercetin and a disease-related protein target. The second category ‘Disease with protein targeted by similar compound’ includes predicted disease associations, where there exist experimental activity data for a compound similar to quercetin (Tc > 0.85) and a disease-related protein. The user has the option to click on one of the results on the right side of the panel, to open in a new tab the respective ChEMBL compound activity data or the protein information in UniProt. The third category ‘Disease with no target information’ includes predicted disease associations with no experimental activity data for quercetin or a structurally similar compound and a disease-related protein.

By clicking on the top right button, ‘Plant disease associations’ the disease network of the query ‘pomegranate’ is displayed, with 15 diseases, by default, in which pomegranate is known to have an effect. When the edge is blue, it indicates a ‘preventive’ association (food associated with disease prevention or amelioration), whereas a red edge indicates a ‘promoting’ association (food associated with disease progress). On the right-hand side, the user can directly access all relevant references in PubMed in support of the pomegranate-disease associations, by clicking on the respective PMID.

### Disease as query

**Figure 3.**
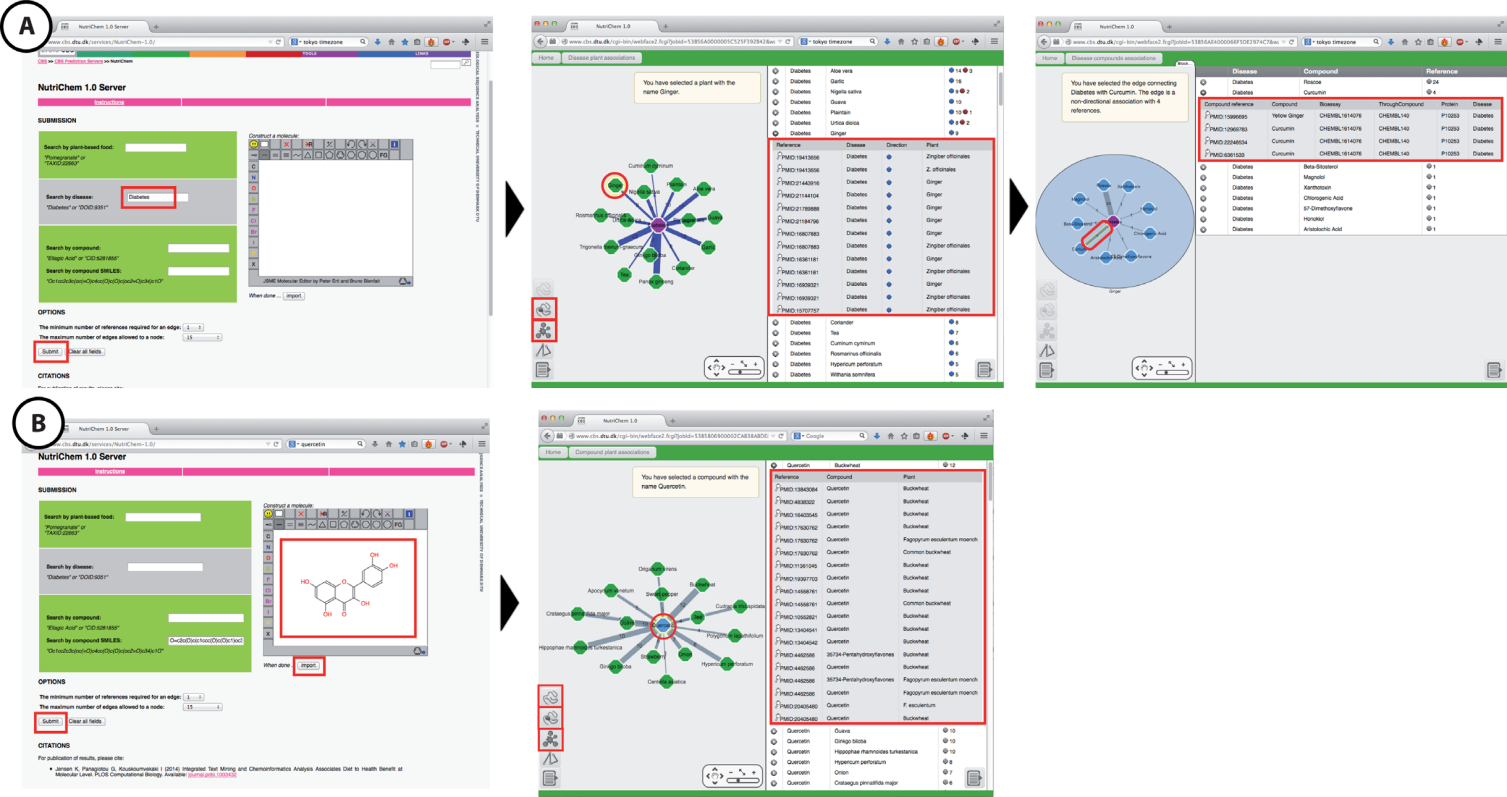
(**A**) Diabetes as query returns, by default, a network of 15 foods, all, in this case, with a preventive association against the disease. On the right-hand side, the user can directly access the relevant references in PubMed in support of all diabetes-food associations, by clicking on the respective PMID. Clicking on any of food names on the network enables the action buttons ‘Get compound activities to disease proteins’ and ‘Get predicted compound activities’. On the right side of the panel the user can click on a circle symbol, which will expand the results and display the experimental activity data related to each compound. The user has again the option to click either on the bioassay, the compound or the protein IDs, which will open in a new tab the respective ChEMBL and UniProt pages. (**B**) Quercetin as query returns, by default, 15 foods that have been associated with it in the literature. On the right-hand side the user can directly access the relevant references in PubMed of all the published quercetin-food associations, by clicking on the respective PMID. Clicking on the compound node enables the three action buttons, as described above.

The user can query ‘diabetes’ which returns, by default, a network of 15 foods that all have, in this case, a preventive association with diabetes (Figure 3A). On the right-hand side the user can directly access all relevant references in PubMed in support of the diabetes-food associations—blue for preventive, red for promoting—by clicking on the respective PMID. Clicking on any of the food names on the network enables the action buttons ‘Get compound activities to disease proteins’ and ‘Get predicted compound activities’, as described above. Ginger, for example, has been associated in the literature with nine compounds with experimental biological activity against a diabetes-related target. On the right side of the panel, the user can click on a circle symbol, which will expand the results and display the experimental activity data related to each compound. For example, by expanding the results for curcumin, we see that it targets P10253 (lysosomal alpha-glucosidase), a clinical trial target against diabetes mellitus type 2 according to TTD (15). The user has again the option to click either on the bioassay, the compound, or the protein IDs, which will open in a new tab the respective ChEMBL and UniProt pages.

### Compound as query

Quercetin as query returns, by default, a network of 15 foods, which have been associated with it in the literature (Figure 3B). On the right-hand side the user can directly access the relevant references in PubMed of all the published quercetin-food associations, by clicking on the respective PMID. Clicking on the compound node enables the three action buttons that allow the use to perform the steps described above.

## CONCLUSIONS

The need for a more accurate assessment of environmental factors in epidemiological studies has given birth to a new ‘ome’, the exposome. Here, we provide a framework for elucidating the link between diet, molecular biological activity and diseases by developing NutriChem, a database source that translates the effect of plant-based food on health from concept to utility. NutriChem relies heavily on the availability of information related to the small molecule constituents of our diet and we plan to maintain and expand it regularly. As the next thing in the pipeline, we intend to integrate in NutriChem biological activity data of marketed drugs, which will make possible to study the effect of diet on drug properties related to their pharmacokinetics and pharmacodynamics.
